# Fascia and Primo Vascular System

**DOI:** 10.1155/2015/303769

**Published:** 2015-08-25

**Authors:** Chun Yang, Yi-kuan Du, Jian-bin Wu, Jun Wang, Ping Luan, Qin-lao Yang, Lin Yuan

**Affiliations:** ^1^Key Laboratory of Optoelectronic Devices and Systems of Ministry of Education and Guangdong Province, College of Optoelectronic Engineering, Shenzhen University, Shenzhen 518060, China; ^2^School of Medicine, Shenzhen University, Shenzhen 518052, China; ^3^Department of Anatomy, Southern Medical University, Guangzhou 510515, China

## Abstract

The anatomical basis for the concept of acupuncture points/meridians in traditional Chinese medicine (TCM) has not been resolved. This paper reviews the fascia research progress and the relationship among acupuncture points/meridians, primo vascular system (PVS), and fascia. Fascia is as a covering, with common origins of layers of the fascial system despite diverse names for individual parts. Fascia assists gliding and fluid flow and holds memory and is highly innervated. Fascia is intimately involved with nourishment of all cells of the body, including those of disease and cancer. The human body's fascia network may be the physical substrate represented by the meridians of TCM. The PVS is a newly found circulatory system; recent increased interest has led to new research and new discoveries in the anatomical and functional aspects of the PVS. The fasciology theory provides new insights into the physiological effects of acupuncture needling on basic cellular mechanisms including connective tissue mechanotransduction and regeneration. This view represents a theoretical basis and means for applying modern biomedical research to examining TCM principles and therapies, and it favors a holistic approach to diagnosis and treatment.

## 1. Introduction

Fascia is connective tissue that surrounds and connects every muscle and organ, forming continuity throughout the body. Traditionally, fascia has been thought of as a passive structure. However, it is now evident that fascia is a dynamic tissue with complex vasculature and innervation [1]. A definition of fascia, especially as an integral tissue has been provided here, highlighting the main features of the superficial fascia [2]. Wide anatomic variations and site-specific differences in fascial structure are described, coupled with results of our extensive investigations of fascia anatomy [3]. The anatomy of the fascia network in human body, as demonstrated through the virtual Chinese human (VCH) research and living body imaging studies, is consistent with the traditional view of the meridians, and the efficacy of acupuncture has been shown to rely on interactions with the fascia [4]. Additionally, it appears that the fascia mediates an active mechanical transference role as it provides dynamic connections between and among the muscles and bones. Moreover, the phenomenon of neurogenic inflammation triggered by stimulation of nociceptive receptors [5] in fascia tissues is consistent with the notion that disruption of fascia physiology can have notable consequences on human health. Indeed, it is our view that neurogenic inflammation in fasciae may constitute a form of disruption of meridian energy flow in traditional Chinese medicine (TCM). Since the first report on the primo vascular system (PVS) by Dr. Bong-Han Kim in 1962, there has been significant progress in this research. The PVS is considered as a newly found circulatory system, which is independent of the blood or lymphatic systems. Identification, harvesting, and characterization of the PVS have been a major challenge due to its small size and transparent optical properties. Over the last decade PVS has attracted interest among researchers both anatomically and histologically. However, it is still unclear what functions primo tissues do.

## 2. Fascia

Fascia is an uninterrupted viscoelastic tissue which forms a functional 3-dimensional collagen matrix [6] and surrounds and connects every muscle and organ, forming continuity throughout the body [7]. It surrounds and penetrates all structures of the body extending from head to toe, thus making it difficult to isolate and develop its nomenclature. Fascia is considered to be any dense irregular connective tissue sheet in the human body, including aponeuroses, joint capsules, or muscular envelopes such as the endo-, peri-, and epimysium. The epimysium surrounds each muscle and is continuous with tendons that attach muscles to bones. The perimysium divides the muscle into fascicles or muscle fiber bundles. The endomysium is a continuous network of connective tissue that covers individual muscle fibers. Small fascial fibers extend to connect to the cell membrane itself.

Fascia is innervated, mostly by proprioceptive nerves. It is intimately involved with the autonomic nervous system as a sensory, target organ [8]. Both myelinated and unmyelinated fibers in fascia were probably of autonomic origin. The outer layers of the limb deep fascia contained a rich vascular and nerve supply with intrafascial nerve fibers throughout the deep fascia. Some researchers also observed Ruffini and Pacini corpuscles, confirming the earlier findings of Yahia et al. in relation to the lumbodorsal fascia. There were also small nerves oriented perpendicularly and attached to the collagen fibers, which presumed to be stretch receptors. And some small nerves displayed morphological characteristics of autonomic nerves.

Fascia is also capable of transmitting electrical signals throughout the body [9]. Collagen, which is one of the main components of fascia, has been shown to have semiconductive, piezoelectric, and photoconductive properties in vitro. Electronic currents could flow over greater distances. These electronic currents within fascia can be altered by external influences and cause a physiologic response in neighboring structures.

All living cells express some inherent contractility by generating tension within their internal cytoskeleton [10]. Fascia also plays a dynamic role in transmitting mechanical tension and may be able to contract in a smooth muscle like manner [9]. Human lumbar fascia can autonomously contract, hypothesized to be due to the presence of contractile cells within fascia. Fibroblasts, which were contained in fascia, can transform into myofibroblasts, express a gene for alpha-smooth muscle actin, and display contractile behavior. The mechanical forces exerted by these cells regulate cytokine synthesis and production of extracellular matrix components and other processes essential to tissue remodeling [11, 12].

Fascia forms a whole-body continuous matrix that interpenetrates and surrounds all organs, muscles, bones, and nerve fibers. It could be considered as a single organ, a unified whole, connection to every aspect of human physiology [13].

## 3. Primo Vascular System

The primo vascular system (PVS) was first reported by Bong-Han Kim in the early 1960s, which is a third vascular compartment in addition to the blood and lymphatic systems. The PVS is optically semitransparent, including several subsystems such as Bonghan corpuscles and Bonghan ducts. The structure was also found by Fujiwara's follow-up. Unfortunately, shortly after Kim's reports in 1960s, the PVS research was suddenly discontinued for the method was not disclosed and the experiments were hard to reproduce. In the 2000s, the research was reinitiated. There have been numerous descriptions of the primo vascular system which is comprised of primo nodes (PN) and primo vessels (PV).

Some researchers also have found primo tissues on the surface of the internal organs of various animals such as mice, rats, rabbits, dogs, swine, and cow, including intestine, cardiac vascular vessels, brain [14, 15], adipose tissue [16], inside the blood [17] and lymphatic vessels, epineurium, running along the sciatic nerve, and below the skin. Lee reported on the observation of a human PVS on both the epithelial fascia and inside the blood vessels of the umbilical cord [18]. The PVS has been distinguished from other similar-looking lymphatic vessels and vascular systems by immunostaining [19]. These findings have led them to consider primo vascular system another circulating system [20]. Moreover, Soh claimed that primo nodes and primo vessels were related to acupuncture points and primo vascular system might be an extension of meridians. PVS exists in most mammalian organs, forming an extensive network throughout the entire body. It is considered as the anatomical basis of classical acupuncture meridians.

In order to confirm the structure of PVS, a lot of labs in Korea, China, and USA tried to repeat the labeling methods of PVS provided by Soh. PVS in enterocelia were identified and stained by dropping 0.2% diluted Trypan blue solution. But PVS cannot be found in all the subjects by the staining method. The percentage of celiac PVS emergence was related to a lot of factors such as age and method of anesthesia. This indicated that the PVS may be related to a pathological process. In conclusion, the emergence of PVS could be affected by age and urethane injection methods. So PVS may not be an intrinsic structure of the body and may be a pathological product which is related to the process of inflammation.

As reported, PVS is also associated with tumor. A cancerous environment triggers cancer PVS formation. And it exists not only in and around tumors [21] but also within the tumors. Islam [22] had provided strong evidence of the existence of a tumor-derived PVS in and around the fascia of tumor xenografts. They all appear to be parallel to their associated neurovascular bundles. Tumor-associated PVS harbors a unique population of tumor-derived cells which express high levels of stem cell specific transcription factors. PVS may play an important role as a stem cell “niche.” In addition, it was shown that PVS connect the primary and secondary tumors and that cancer cells were transported via the PVS in an active manner [23]. It has been suggested that the PVS might contribute to tumor growth and metastasis [21]. PVS may also be a unique “niche” for cancer stem cells. The locations of the PVS floating in fluid are not fixed, and those fixed-location PVS like intraorgan PVS are not yet observed. The origin of the primo vessels and nodes associated with xenografted tumor is the host animal, but cells like the histiocytes in the primo node are from the tumor.

In 2000s, Park first started to measure resting and spontaneous potentials in primo node. More researchers have studied PVS electrophysiological characteristics after this study, and they have found bioelectrical signals from primo vessels and lymphatic vessels were different. The small intestine and lymphatic vessels generate an action potential to transfer materials. Neuron spikes are generated when neurons exchange electrical signals. PVS perform different functions in smooth muscles and neurons. The researchers speculate that PVS transfer signals in distinct manners for neurons and do not directly move materials, such as through the small intestine and lymphatic vessels. PVS was considered the substances in acupoints and meridians.

So far, the specific function of PVS in biological processes remains unclear. As reported, the structure of the PVS is distinct from the well-known tissues such as nerves and blood vessels and may be related to acupuncture meridian and the acupuncture points of TCM. The PVs in surface of internal organs did not have an effect in regulating gastric motility induced by acupuncturing at CV12 nor in the facilitation of gastric motility induced by acupuncturing at ST36. The results are valid for the subclass of PVS on the surface of internal organs (OS-PVS). There is a complicated network of five subclasses of PVS, and the most important ones with respect to the intestinal motility are those along blood vessels and nerves as implied in Kim's work. The OS-PVS is deeply related to stem cell like functions and immune functions. It is timely that functional aspects of the PVS are to be studied with respect to both Western and Eastern medicines.

## 4. Fasciology

According to Professor Lin Yuan, there are close relations of the meridians and acupoints to connective tissues. Under the VCH project, we marked the regions rich in connective tissues on the tomographic images for three-dimensional (3D) reconstruction of the whole-body fascia framework, and the established digital model showed an approximate match with the distribution of the meridian and acupoints. The acupoints were found to locate mainly at the sites with enrichment of certain connective tissues, such as the muscular septa of the limbs, structures with abundant somatic nerve endings, the internal organs with rich sensory nerve distribution, and the visceral mesenteries [24, 25]. All the marked fascia in the body constituted a complete body-shaped framework, and we therefore hypothesized that the fascia network was the anatomical basis of the meridians.

To explore the theoretical support of our hypothesis, Professor Lin Yuan examined developmental biology, the developmental process of an individual embryo, and the evolution of fascia. The fascia network was derived from the residual mesenchyme after it had differentiated into different organs or systems. The extracellular matrix of a single germ layer organism, the mesogloea of a two-germ-layer organism, the mesenchyme of a three-germ-layer organism, and the nonspecific connective tissue of the human body are all homologous structures. The nonspecific connective tissue network in the human body provides cell storage and maintains the stability of the internal environment by cell proliferation, differentiation, repair, and regeneration. We therefore established a new anatomical approach from a dynamic point of view and proposed the two-system theory. In light of this theory, the human body can be divided into two systems. One is the supporting-storing system, consisting of undifferentiated nonspecific connective tissue. The other is the functional system, consisting of various differentiated functional cells. Based on this theory, we can further explore a new research area, fasciology [2]. The term fasciology indicates a biomedical orientation of the TCM theory. According to fasciology, from the axis in the absence of biological life in Darwin's theory of evolution to the Yellow Emperor's understanding of the life axis, we transform the present biomedical research in the two-dimensional coordinates into more complex three-dimensional coordinates.

### 4.1. Two-System Theory

In the two-system theory, the supporting-storing system consists of undifferentiated cells in unspecialized connective tissues, and the functional system contains diverse differentiated functional cells supported or surrounded by the supporting-storing system. The undifferentiated stem cells in the supporting-storing system constantly differentiate into functional cells. The supporting-storing system throughout the body regulates the functionality and life activities of the differentiated cells and provides a stable environment for their survival. In this context, we put forward a new approach to the division of the discipline anatomy. The anatomical discipline based on the two-system theory is fascial anatomy and studies the human body in light of how organisms survive with a longer life cycle, which is different from regional anatomy study that examines anatomical structures and systematic anatomy that study the functions.

### 4.2. Fascial Anatomy

Fascial anatomy is a new perspective on anatomy. It classifies body structures into the supporting-storing system and the traditional functional system. This perspective is also applicable to all the living organisms, from a primitive unicellular organism to a higher mammal. It studies the morphological transformation during evolution from simple to complex organisms. It also investigates how an organism maintains a longer life span through the evolution of the supporting-storing system.

Fascia anatomy studies the structure of an organism based on the two-system theory. Fascia anatomy is different from traditional regional anatomy and systematic anatomy. Regional anatomy only studies local human structures and systematic anatomy studies the human body on both a morphological and functional basis. Fascial anatomy incorporates a third parameter, time, to study not only the structures and functions of the body but also the morphological transformation during evolution and embryonic development. It investigates how an organism, such as a primate, can maintain a longer life span through evolution of the supporting-storing system from the mesoderm. Therefore, fascia anatomy helps scientists better understand the biological essence of an organism by reminding them to study anatomy in a dynamic perspective; that is, all cells and organs maintain their normal structures and functions through the interaction between the supporting-storing system and the functional system.

In other words, fascial anatomy switches anatomical study from the “dead” to the “living.” When the supporting-storing system wears out, the body will die. When the wax is depleted, the flame will extinguish, as with the human body.

## 5. The Relationship between Meridians and Fascia

The theory of meridians and collaterals is a fundamental pillar of TCM, particularly in the areas of acupuncture, moxibustion, and massage, as well as of traditional martial arts such as Tai Chi Chuan. Meridians are essentially strings of acupoints, which may be visualized as passageways through which energy flows throughout the body. The anatomical basis of acupuncture stimulation is the fascia (like intermuscular septum and intermuscular space), which can generate strong biological information upon rotation of the needles [26]. There is only a quantitative, but not qualitative, difference between the acupoints and nonacupoints in the biological information they produce [27]. Similarly, the Chinese herbal medicine regulates the regeneration and activity of the functional cells by improving the microcirculation and permeability of epithelial basement membrane in the fascia.

Fascia has specific cells, ground substance, and fiber types that make it a form of connective tissue proper. A better understanding of fascia at the cellular level gives insight into its functional properties. The cells within fascia include fibrocytes (fibroblasts, myofibroblasts), adipocytes, and various migrating white blood cells [28]. The fascia network of collagen and ground substance is maintained by fibrocytes. Fibrocytes regulate interstitial fluid volume and pressure as well as the extracellular molecular components [6]. It also responds to mechanical stretch through mechanotransduction. Langevin verified the mechanism of mechanotransduction in vivo that applied mechanical stress induces a change in cell morphology. Barnes notes that when performing myofascial release the response is felt in 90–120 s, and therefore any matrix adaptations initiated by a change in mechanical stress apparently take too long to occur to explain the observed immediate benefits of mechanical therapies. Fibrocytes may further transform themselves into myofibroblasts through this mechanical tension, as observed in wound healing [29]. However, myofibroblasts also appear to be a normal component of fascia and importantly they are also observed additionally in epimysium and perimysium. The contractile nature of these cells appears to give them ability to alter tissue tension, through contraction and relaxation, in the short timescales observed in practice.

Steven Finando reconsiders acupuncture, positing that the fascia is the mechanism of action of acupuncture therapy. The fascia has also been conceived as a complex communication network that influences and is influenced by every muscle, organ, blood vessel and nerve. Langevin suggests the fascia to be a metasystem, connecting and influencing all other systems. Incorporating this view would change our core understanding of human physiology. The cytoskeleton of fascia cellular under continuous tension is capable of transmitting mechanical forces through the fascia system. Forces applied to the cytoskeleton can produce biochemical changes on the cellular level by mechanochemical transduction [30]. Guimberteau demonstrates the complex fractal structure of the tissue and how it allows for movement, adaptation, lubrication, and repair. The fascia as our richest sensory organ permeated with four types of sensory receptors. The vascular, nervous, and lymphatic systems all end in the ground substance, providing nutrients to the ground substance as well as information from the periphery. It is both interesting and highly significant to note that acupuncture is based upon the conception of a metasystem that links and influences every aspect of human physiology. The fascia system provides the anatomical basis of that metasystem.

Acupuncture needle manipulation causes mechanical deformation of connective tissue, which in turn results in mechanical stimulation of fibroblasts, with active changes in cell shape and autocrine purinergic signaling [31]. The biomechanical behavior of connective tissue in response to stretching is generally attributed to the molecular composition and organization of its extracellular matrix. It also is becoming apparent that fibroblasts play an active role in regulating connective tissue tension. In response to static stretching of the tissue, fibroblasts expand within minutes by actively remodeling their cytoskeleton. This dynamic change in fibroblast shape contributes to the drop in tissue tension that occurs during viscoelastic relaxation.

The PVS is a novel circulatory system forming a network throughout an animal's body. Bong-Han Kim identified the novel anatomical vessels as meridian primo vessels and proposed that their distribution mirrored acupuncture meridians. According to Dr. Soh's observation, the histological structures of PVS and PN are abundant collagen fibers and elastic fibers. These fibrillar materials are composed of thread-like structures suggestive of collagen and/or elastic fibers. Because of the abundant connective tissue fibers, it could explain why excised vessels and nodes are very elastic in nature and have a tendency to coil spontaneously [32]. The connective tissue is the carrier of the mechanical stimulation induced by acupuncture. According to Professor Kim's conception, all the nuclei of tissue cells are connected with fine terminal subducts, which are connected to the primo vessels for the organs. Acupuncture may regulate organs' function by simulating exterior PVS and PNs through the exterior tissue cells. As reported, fibroblasts and leukocytes might be two kinds of cell types in PVS for both of infected and untreated rats [22]. The suggested functions of the PVS, in general, include a path for neurotransmitter hormones, a circulatory path for primo fluid-containing stem cell like microcells, and proteins related to stem cell differentiation. Evidence also exists for cancer metastasis through the primo vessel. Moreover, PNs and PVS were related to acupuncture points and primo vascular system might be an extension of meridians. Only by establishing the functional connection of the exterior-interior PVS between the stimulus of acupoints and responses of organs could PVS be a basis for meridians. Thus, a comparison between acupuncture meridians and PVS leaves nothing rigorous but a mist. The data demonstrate that PVS is a novel and distinctive structure, but the criteria of it are still needed to develop. We pay more attention to the function of PVS related to meridians. The study with PVS on the organ surface showed that they are not involved with acupuncture stimulations, and further studies with skin PVS and extra PVS are required to find out the functional relation with acupuncture.

The PVS is thought to originate from fascia connective tissue and be developmentally mesodermal in origin. The PVS is an anatomical structure corresponding to the acupuncture meridians and the acupuncture points of TCM. Meridians have been considered as a part of fascia and the “fasciology” theory used to explain the physiology of acupuncture in general. But the function of the PVS with respect to nerve regeneration and acupuncture is not yet studied. With the deep research of the fasciology and PVS, there will be a bright future of the TCM research.
